# The Intersection of Heart Failure and Chronic Kidney Disease: Challenges in Co-management

**DOI:** 10.7759/cureus.100993

**Published:** 2026-01-07

**Authors:** Amaan Alvi, Hithyshi Singamala, Sai Surya Vamsi Gadde, Chaitanya Kumar Javvaji

**Affiliations:** 1 Internal Medicine, King George's Medical University, Lucknow, IND; 2 Internal Medicine, Apollo Institute of Medical Sciences and Research, Chittoor, IND; 3 Internal Medicine, Gayatri Vidya Parishad Institute of Health Care and Medical Technology, Visakhapatnam, IND; 4 Pediatrics, Jawaharlal Nehru Medical College, Datta Meghe Institute of Higher Education and Research, Wardha, IND

**Keywords:** cardiorenal syndrome, finerenone, heart failure, hyperkalemia, mineralocorticoid receptor antagonists, nt-probnp

## Abstract

Heart failure (HF) and chronic kidney disease (CKD) frequently coexist, creating a bidirectional relationship where dysfunction of one organ accelerates deterioration of the other. This overlap is associated with high morbidity, mortality, and healthcare costs. Yet, patients with advanced CKD are systematically underrepresented in pivotal HF trials, resulting in major evidence gaps and therapeutic uncertainty. The pathophysiological interplay involves hemodynamic congestion, activation of the renin-angiotensin-aldosterone system (RAAS) and sympathetic nervous system (SNS), oxidative stress, inflammation, and nontraditional mediators such as uremic toxins and gut-derived metabolites, including trimethylamine-N-oxide (TMAO). Diagnosing HF in the context of CKD is challenging due to overlapping clinical features, limitations of biomarkers such as B-type natriuretic peptide (BNP) and N-terminal pro-B-type natriuretic peptide (NT-proBNP), and imaging constraints. At the same time, treatment is complicated by risks such as hyperkalemia, worsening renal function, and altered drug pharmacokinetics. Although guideline-directed medical therapies, including RAAS inhibitors, beta-adrenergic receptor blockers (beta-blockers), mineralocorticoid receptor antagonists (MRAs), and sodium-glucose cotransporter-2 (SGLT-2) inhibitors improve outcomes in HF, their use in advanced CKD is limited by safety concerns and lack of trial data. Recent advances include the nonsteroidal MRA finerenone and SGLT-2 inhibitors, which have demonstrated cardiovascular and renal benefits in patients with mild to moderate CKD, as well as acetazolamide, which has shown short-term efficacy as an adjunctive decongestive therapy in acute HF. However, the safety, efficacy, and clinical relevance of these approaches remain insufficiently defined in advanced CKD and in patients receiving dialysis. Multidisciplinary care models, precision medicine approaches, and digital innovations such as telemedicine and artificial intelligence-driven risk prediction promise to overcome fragmented management and improve patient-centered outcomes. This review highlights current challenges, synthesizes emerging evidence, and outlines future directions for optimizing the co-management of this complex and high-risk population.

## Introduction and background

Heart failure (HF) and chronic kidney disease (CKD) frequently coexist, forming a bidirectional relationship where dysfunction of one organ worsens the other. This overlap contributes to high morbidity, mortality, and healthcare costs [1]. Yet patients with advanced CKD are often excluded from pivotal HF trials, creating evidence gaps. Renal dysfunction complicates up to half of HF hospitalizations [2] and is a strong predictor of poor outcomes. Pathophysiologically, neurohormonal activation, venous congestion, oxidative stress, and gut microbiome metabolites like trimethylamine-N-oxide (TMAO) drive the vicious cycle of cardiorenal syndrome (CRS) [3-6]. Diagnosis is hampered by overlapping signs and biomarker limitations, requiring integration of imaging and clinical context. Therapy is constrained by hyperkalemia risk, reluctance to start or titrate therapies, and lack of trial data in advanced CKD.

Epidemiology and prevalence

CKD affects an estimated 9-16% of adults worldwide, with prevalence increasing sharply with age [7]. The coexistence of HF and CKD is common and bidirectional, as each condition exacerbates the other. Declining renal function, particularly when the estimated glomerular filtration rate (eGFR) falls below 60 mL/min/1.73 m², significantly increases the risk of developing HF [8]. Conversely, in patients hospitalized with HF, renal dysfunction is present in one-third to half of cases and strongly predicts adverse outcomes, including rehospitalization and mortality [9]. Large-scale epidemiologic studies confirm that CKD confers heightened risks of death, cardiovascular events, and hospital admissions [10,11]. These shared burdens underscore the difficulties in managing coexisting HF and CKD, as treatment strategies for one condition often affect the other, complicating care optimization for this high-risk population.

This review is particularly timely given the rising global burden of multimorbidity, with HF and CKD among the fastest-growing chronic conditions that frequently overlap [7,12]. Despite their prevalence and shared mechanisms, patients with advanced CKD remain systematically underrepresented in pivotal HF randomized controlled trials, creating significant evidence gaps and therapeutic uncertainty. By focusing on the co-management challenges of this dual disease state, this review synthesizes emerging insights, highlights barriers in real-world implementation, and outlines pragmatic strategies for optimizing care.

Impact on morbidity and mortality

Renal impairment continues to be a significant, independent predictor of adverse outcomes in HF, with CKD present in over 50% of HF patients and exerting particularly strong effects in heart failure with reduced ejection fraction (HFrEF) compared to heart failure with preserved ejection fraction (HFpEF). HF hospitalizations in CKD patients are associated with high morbidity and mortality. The interplay of CKD and HF creates a negative spiral, as each condition accelerates the other [1].

Challenges in co-management, diagnostic complexity, treatment gaps, and evidence limitations

Many pivotal HF trials excluded patients with advanced CKD. As a result, guideline-directed medical therapies are underutilized in this group [13]. Beta-blockers are supported across CKD stages, including dialysis, though high-quality evidence is limited [14]. Sacubitril-valsartan shows benefit in HF, but its use in advanced CKD remains nuanced [15,16]. Sodium-glucose cotransporter-2 (SGLT2) inhibitors (dapagliflozin, empagliflozin) demonstrate strong cardiovascular and renal benefits, though advanced CKD patients are still underrepresented in major trials [17]. Nonsteroidal mineralocorticoid receptor antagonists (MRAs), such as finerenone, have shown cardiorenal benefit in patients with CKD and type 2 diabetes, but HF-specific evidence is still emerging [18]. Diagnosing and managing HF-CKD overlap is challenging due to shared symptoms such as fluid overload and confounding effects on biomarkers [19,20]. Polypharmacy and conflicting guidance across specialities often hinder consistent, coordinated care [21].

Interdisciplinary HF-CKD clinics show promise but remain limited in availability. Digital innovations such as artificial intelligence, telemedicine, and remote monitoring are emerging but not yet standard practice [22]. Fluid management tools (e.g., lung ultrasound, ultrafiltration, and peritoneal dialysis) can support tailored decongestion strategies [15]. Dietary interventions, such as sodium restriction, demonstrate cardiovascular benefit in CKD, though optimal targets remain debated [23]. The high coexistence, poor outcomes, and therapeutic underutilization highlight the urgency of addressing HF-CKD overlap. Tackling fragmented care models and treatment hesitancy may improve survival, reduce hospitalizations, and enhance quality of life. Multidisciplinary care, precision medicine, digital health, and emerging therapies offer promising pathways to advance the management of this complex, high-risk population [24].

This narrative review was performed by a targeted, non-systematic search of the medical literature. Relevant articles were identified through searches of PubMed and Google Scholar, focusing on studies published primarily within the past 10-15 years. Priority was given to major clinical practice guidelines, landmark randomized controlled trials, large observational studies, meta-analyses, and high-quality review articles addressing the intersection of HF and CKD. Additional references were identified through manual screening of bibliographies from key publications. Evidence was selected and synthesized based on clinical relevance, methodological quality, and applicability to different HF phenotypes and stages of CKD. Formal risk-of-bias assessment was not performed, consistent with the narrative scope of this review.

## Review

Pathophysiological overlap between HF and CKD

Hemodynamic Dysregulation

In HF, elevated central venous pressure (CVP) and intra-abdominal pressure impair renal perfusion and reduce glomerular filtration rate (GFR), often exerting a greater impact than reduced cardiac output [25]. These hemodynamic stresses cause renal venous congestion, sodium retention, and progressive worsening of kidney function [26]. Fluid overload in HF, therefore, feeds back to the kidneys, raising venous pressures and further impairing filtration [25].

Neurohormonal Activation

Neurohormonal activation in HF and CKD reflects a complex, multidimensional process rather than a simple consequence of reduced cardiac output or renal hypoperfusion. Although impaired forward flow may contribute to advanced systolic dysfunction, growing evidence indicates that venous congestion, elevated central and renal venous pressures, and disruption of effective arterial blood volume are key drivers of renin-angiotensin-aldosterone system (RAAS) and sympathetic nervous system (SNS) activation [25,26]. Increased renal venous pressure reduces transglomerular perfusion gradients, promotes sodium and water retention, and sustains maladaptive neurohormonal signaling even in patients with preserved or mildly reduced ejection fraction, in whom cardiac output is often maintained [1]. Importantly, RAAS and SNS activation may persist despite correction of forward flow, highlighting that hypoperfusion alone is insufficient to explain ongoing sodium retention, fibrosis, and progressive remodeling [27]. In addition to hemodynamic factors, non-hemodynamic mechanisms, including chronic inflammation, oxidative stress, and altered autonomic and baroreceptor signaling, further amplify neurohormonal tone in both HF and CKD [1,28]. Through these converging pathways, angiotensin II and aldosterone promote myocardial and renal fibrosis, endothelial dysfunction, and progressive volume dysregulation across diverse HF phenotypes, reinforcing the self-perpetuating nature of cardiorenal dysfunction [27].

Oxidative Stress and Inflammation

Chronic RAAS and SNS activation increases reactive oxygen species (ROS) and systemic oxidative stress. Inflammatory cytokines such as tumor necrosis factor-alpha (TNF-α), interleukin-6 (IL-6), tumor necrosis factor-like weak inducer of apoptosis (TWEAK), and C-reactive protein (CRP) are elevated in HF and CKD and drive endothelial dysfunction, fibrosis, and organ injury [1,28].

Uremic Toxins and Nontraditional Risk Mediators

Protein-bound uremic toxins (indoxyl sulfate, p-cresyl sulfate) induce oxidative stress, endothelial dysfunction, and fibrosis in both organs [21]. Fibroblast growth factor-23 (FGF-23) is elevated in CKD and promotes left ventricular hypertrophy and remodeling [29]. Additional nontraditional risk mediators include gut-derived metabolites such as TMAO, which has been linked to higher cardiovascular risk and CKD progression [30].

Gut Microbiome, TMAO, Anemia, and Microvascular Inflammation

Emerging evidence implicates gut microbiome-derived metabolites, particularly TMAO, as potential contributors to the cardiorenal axis; however, the strength and nature of this relationship require careful interpretation. Observational cohort studies consistently demonstrate associations between elevated circulating TMAO levels and adverse cardiovascular and renal outcomes, including incident HF and progression of CKD, independent of conventional risk factors [30]. Importantly, these findings are primarily associative, and in advanced CKD, reduced renal clearance likely contributes substantially to TMAO accumulation, raising the possibility of reverse causation rather than a strictly upstream pathogenic role. Experimental and preclinical studies suggest that TMAO may promote vascular inflammation, endothelial dysfunction, and myocardial fibrosis, but the direct relevance of these mechanisms to human HF phenotypes and stages of CKD remains incompletely established [30]. In parallel, microvascular dysfunction, affecting both coronary and renal microcirculatory beds, has been linked to chronic inflammation, oxidative stress, and endothelial injury in HF and CKD, and may act as both a mediator and a marker of disease severity rather than a unidirectional driver of myocardial injury [1,28]. Anemia, a frequent comorbidity in both conditions, is likewise heterogeneous in origin, encompassing iron deficiency, erythropoietin deficiency, inflammation-mediated functional iron deficiency, and hemodilution. Across these subtypes, anemia is associated with reduced oxygen delivery, heightened neurohormonal activation, and worse clinical outcomes, although the relative contribution of each mechanism varies across disease stage and patient phenotype [31]. Collectively, these nontraditional mediators highlight important biologically plausible pathways within the cardiorenal continuum, while underscoring the need for cautious interpretation of causality and greater mechanistic precision.

Classification and mechanistic insights of cardiorenal syndrome (CRS)

CRS encompasses five subtypes, each defined by the primary organ affected and the acuity of dysfunction. Type 1, or acute CRS, refers to acute HF causing acute kidney injury through mechanisms such as hypoperfusion, congestion, and neurohormonal activation. Type 2, or chronic CRS, involves chronic HF progressively impairing kidney function due to sustained hemodynamic and neurohormonal changes. Type 3, or acute reno-cardiac syndrome, occurs when acute kidney injury triggers acute cardiac dysfunction through systemic inflammation, electrolyte and acid-base imbalances, and the accumulation of uremic toxins. Type 4, or chronic reno-cardiac syndrome, describes CKD driving cardiac dysfunction via mechanisms, including uremic toxins, vascular calcification, anemia, and chronic inflammation. Finally, type 5, or secondary CRS, results from systemic illnesses such as sepsis, diabetes, cirrhosis, or lupus that simultaneously impair both the heart and kidneys through pathways including cytokine storm, RAAS, and complement activation, and circulatory disturbances [32,33]. Table 1 summarizes the classification of CRS.

**Table 1:** Classification of cardiorenal syndrome (CRS) along with triggers, onset, and key pathophysiological mechanisms. RAAS: renin–angiotensin–aldosterone system; SNS: sympathetic nervous system; CKD: chronic kidney disease; CRS: cardiorenal syndrome. Table credit: Hithyshi Singamala References: [32,33]

CRS type	Primary trigger	Onset	Pathophysiological mechanisms
Type 1 – Acute cardiorenal	Abrupt cardiac dysfunction → acute kidney injury	Acute → Acute	Renal hypoperfusion or congestion; elevated central venous/intra-abdominal pressure; neurohormonal activation (RAAS, SNS); inflammation; diuretic resistance
Type 2 – Chronic cardiorenal	Chronic cardiac dysfunction → progressive CKD	Chronic → Chronic	Sustained reduced renal perfusion; chronic neurohormonal activation (RAAS, SNS); remodeling and fibrosis
Type 3 – Acute renocardiac	Acute kidney injury → acute cardiac dysfunction	Acute → Acute	Mitochondrial dysfunction; inflammation; oxidative stress; apoptosis; SNS & RAAS activation; inflammasome; uremic toxins; fluid overload; electrolyte/acid–base disturbances
Type 4 – Chronic renocardiac	Chronic kidney disease → chronic cardiac dysfunction	Chronic → Chronic	Atherosclerosis; cardiac hypertrophy; neurohormonal and inflammatory alterations; traditional and nontraditional cardiovascular risk factors
Type 5 – Secondary CRS	Systemic disease → simultaneous heart & kidney dysfunction	Acute or Chronic → Acute or Chronic	Shared insults, including sepsis, diabetes, autoimmune disorders (e.g., lupus), cirrhosis, and amyloidosis; microcirculatory disturbances; high catecholamine/toxin exposure; systemic inflammation [33]

The vicious feedback loop between heart and kidney dysfunction

**Figure 1:**
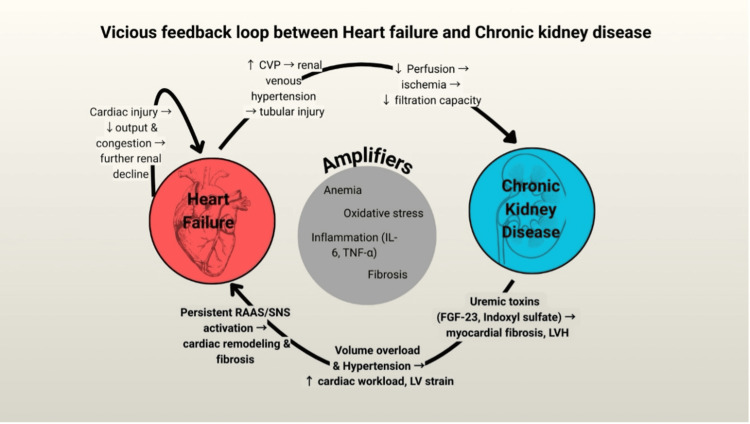
The vicious feedback loop linking heart failure and chronic kidney disease. The figure illustrates central venous pressure-driven renal congestion, decreased renal perfusion and eGFR decline, accumulation of uremic toxins (indoxyl sulfate, p-cresyl sulfate), inflammation and endothelial dysfunction, myocardial fibrosis, and worsening cardiac output, producing a self-perpetuating cycle of cardiorenal deterioration. CVP: central venous pressure; eGFR: estimated glomerular filtration rate; RAAS: renin–angiotensin–aldosterone system; SNS: sympathetic nervous system; TNF-α: tumor necrosis factor-alpha; IL-6: interleukin-6; FGF-23: fibroblast growth factor-23; LV: left ventricle; LVH: left ventricular hypertrophy. Figure credit: Amaan Alvi

The interplay between HF and CKD establishes a self-perpetuating cycle that accelerates disease progression. In HF, elevated CVP and reduced renal perfusion contribute to kidney dysfunction. In turn, CKD promotes neurohormonal activation, accumulation of uremic toxins, and persistent inflammation, all of which drive myocardial injury. This cardiac injury reduces cardiac output and worsens systemic congestion, leading to further decline in renal function. Additionally, anemia and oxidative stress exacerbate dysfunction in both the heart and kidneys, compounding the cycle of deterioration. Figure 1 represents the vicious feedback loop linking HF and CKD.

Diagnostic complexities in coexisting HF and CKD

The coexistence of HF and CKD creates major diagnostic challenges that complicate management. Registry data confirm a high prevalence of renal impairment in HF, with reduced eGFR associated with increased hospitalization, cardiovascular events, and mortality [24,34-37]. The relationship is bidirectional: CKD promotes volume retention, neurohormonal activation, and hypertension, while impaired cardiac output and venous congestion worsen renal perfusion and accelerate kidney injury [1,38]. At presentation, it is often difficult to determine which organ initiated decompensation.

Clinical features overlap substantially. Fatigue, dyspnea, and edema may reflect cardiac pump failure, impaired renal clearance, or both. Volume overload may arise from reduced cardiac output, impaired sodium excretion, or a combination. CKD comorbidities, such as anemia, electrolyte imbalances, and hypertension, can mimic or obscure typical HF signs [39-41]. For this reason, objective testing is essential: biomarkers, imaging, and serial assessments interpreted in context improve diagnostic accuracy and guide therapy.

Limitations of Biomarkers in CKD

Natriuretic peptides like B-type natriuretic peptide (BNP) and N-terminal pro-B-type natriuretic peptide (NT-proBNP) are sensitive markers of wall stress. Still, they are chronically elevated in CKD, even without acute HF, due to reduced clearance and CKD-associated remodeling [42-46]. Thus, conventional cutoffs lose specificity in advanced CKD; trends, relative changes, and higher thresholds may improve diagnostic accuracy. Similarly, high-sensitivity cardiac troponins are frequently elevated chronically in CKD. In this context, distinguishing acute myocardial injury requires serial testing, correlation with symptoms, electrocardiogram changes, and imaging [47,48].

Role of Imaging and Functional Assessment

Echocardiography remains first-line because of accessibility and ability to evaluate systolic/diastolic function, chamber size, valvular disease, and filling pressures. However, CKD-related concentric hypertrophy and fibrosis can complicate interpretation, and left ventricular ejection fraction (LVEF) alone may miss subtle dysfunction; advanced measures such as strain imaging are often helpful [49-52]. Cardiac magnetic resonance (CMR) imaging offers superior tissue characterization, but gadolinium contrast is restricted in advanced CKD due to risks of nephrogenic systemic fibrosis and gadolinium retention. Non-contrast CMR protocols are increasingly feasible and may be considered when available [53-56]. The CRS concept emphasizes this interplay: acute HF can precipitate acute kidney injury (CRS type 1), while primary kidney injury may trigger acute cardiac dysfunction (CRS type 3). Chronic and mixed forms are common [31,32,57]. Identifying the predominant driver during acute episodes is critical to determine priorities - whether to emphasize diuresis, inotropic support, or renal replacement.

Laboratory Assessment and the Importance of EGFR, Albuminuria, and Emerging Biomarkers

Serum creatinine and eGFR remain routine but can be misleading. Fluid overload may dilute creatinine and underestimate renal dysfunction, whereas aggressive decongestion may cause a transient creatinine rise that does not necessarily indicate structural injury [58-62]. Alternative markers such as cystatin C and tubular injury biomarkers may clarify function or detect early injury, but their routine role remains investigational and varies across institutions [63-67].

eGFR and albuminuria are fundamental for risk stratification but must be interpreted in the HF context. Novel biomarkers (e.g., galectin-3) and multimarker panels hold promise for refining diagnosis and prognosis, especially when combined with imaging and risk scores. Multidisciplinary collaboration among cardiology, nephrology, and allied care teams reduces misinterpretation of laboratory fluctuations during therapy and supports safer decision-making. Continued research into biomarker panels, non-contrast imaging, and risk stratification algorithms is needed to improve diagnostic accuracy and outcomes in HF-CKD [57,62,68-70].

Therapeutic dilemmas in dual disease management

Acute decompensation in patients with concomitant HF and CKD rarely reflects a single predominant pathophysiological driver. Instead, congestion, impaired effective arterial blood volume, neurohormonal activation, and intrinsic renal injury commonly coexist and evolve dynamically over time, consistent with the contemporary framework of CRS [1,31-33]. Clinical and laboratory markers frequently used to infer predominance, such as changes in serum creatinine, urine output, blood pressure, or natriuretic peptide levels, are imprecise, may lag behind underlying pathophysiological processes, and are influenced by ongoing therapeutic interventions [57]. As a result, management priorities in acute CRS are guided by an integrated assessment of congestion, perfusion, and metabolic stability rather than attribution to a single dominant mechanism. Therapeutic strategies such as diuresis, vasoactive support, or renal replacement therapy differ substantially in indication, risk profile, and evidence base, and are typically applied in a complementary and iterative manner with close reassessment of clinical and hemodynamic response [1,40]. This dynamic approach better reflects contemporary clinical practice and acknowledges the overlapping and bidirectional nature of acute heart-kidney interactions.

This situation is further complicated by changes in drug pharmacokinetics and a tendency toward electrolyte imbalances, such as hyperkalemia, which can diminish therapeutic effectiveness and increase the likelihood of adverse reactions. Consequently, treatment plans must be carefully customized to the particular HF phenotype (e.g., reduced versus preserved ejection fraction) and the severity of CKD. This highlights the importance of a multidisciplinary team approach and rigorous patient monitoring. Nevertheless, a series of recent landmark studies has started to fill this evidence gap, significantly transforming the treatment landscape for individuals with CRS.

RAAS Inhibitors

RAAS inhibition is a cornerstone of therapy for HFrEF [71]. Angiotensin-converting enzyme (ACE) inhibitors, angiotensin II receptor blockers (ARB), and the newer angiotensin receptor-neprilysin inhibitors (ARNI) consistently improve survival and reduce hospitalizations by limiting maladaptive remodeling and neurohormonal overactivation. In patients with CKD, however, this class presents distinct therapeutic challenges. RAAS blockade dilates the glomerular efferent arteriole, often producing an acute, reversible decline in eGFR [72]. This predictable hemodynamic effect is frequently misinterpreted as intrinsic kidney injury, prompting unnecessary withdrawal. The main adverse effects (hyperkalemia and hypotension) are amplified in CKD, where reduced aldosterone activity further impairs potassium handling [73]. Angioedema remains a rare but serious safety concern with ACE inhibitors, requiring a 36-hour washout before initiating an ARNI [74].

In routine practice, these risks fuel clinician hesitation. Fear of hyperkalemia, misinterpretation of creatinine rises, and therapeutic inertia all contribute to underuse of RAAS inhibitors in the very population that might benefit most [13]. Because patients with eGFR <30 mL/min/1.73 m² and those on dialysis were systematically excluded from pivotal trials, evidence is lacking to reassure clinicians at the extremes of CKD [75]. Until dedicated studies are conducted, real-world management must balance proven survival benefits against perceived risks, emphasizing proactive monitoring, mitigation strategies, and patient-specific decision-making.

Beta Blockers

Beta-adrenergic blockers are a fundamental element of guideline-directed medical therapy (GDMT) for HFrEF [13]. They improve survival and reduce hospitalizations by counteracting chronic SNS overactivation [76-78]. In patients with advanced CKD, however, their use presents important challenges. Pharmacokinetics vary by drug: hydrophilic beta-blockers that undergo renal clearance may accumulate when kidney function declines, increasing the risk of adverse effects such as bradycardia, hypotension, and profound fatigue [79]. These side effects may be particularly poorly tolerated in frail cardiorenal patients. Among individuals on dialysis, therapy becomes even more complex. The dialyzability of different beta-blockers varies; for example, atenolol and metoprolol are readily removed by dialysis, while carvedilol is not, leading to unpredictable drug levels and hemodynamic responses [80]. This variability complicates dosing and contributes to uncertainty in routine practice.

Although observational studies suggest beta-blockers may retain benefits across CKD stages, including dialysis, the absence of robust randomized trial evidence in advanced CKD leaves clinicians uncertain about their net risk-benefit profile. This gap fosters therapeutic caution, underuse, and wide variation in prescribing practices. Until dedicated trials are performed, careful selection of agents, individualized dosing, and close monitoring remain essential for safely extending the benefits of beta-blockade to patients with HF and advanced CKD.

SGLT2 Inhibitors

SGLT2 inhibitors represent a major advance in managing HF and CKD, with consistent benefits across the spectrum of disease and independent of diabetes status [17,81,82]. Unlike other classes of GDMT, the primary challenge with SGLT2 inhibitors is not frequent adverse effects but rather ensuring their broad and consistent adoption while managing a small number of recognizable risks.

The most common side effect is an increased risk of genital mycotic infections, related to glycosuria, though importantly, this does not translate into higher rates of urinary tract infections [83]. A more serious but uncommon complication is euglycemic diabetic ketoacidosis (DKA), which requires clinician awareness and patient education regarding “sick day” rules. Early concerns about increased fracture risk have been refuted by large meta-analyses, which found no meaningful elevation in fracture incidence [84]. Another frequent source of clinical hesitation is the transient dip in eGFR observed after initiation [85]. This change reflects a favorable hemodynamic adjustment (reduced intraglomerular pressure) and predicts long-term kidney protection. However, it is often misinterpreted as acute kidney injury, leading to premature discontinuation. Misunderstanding this expected effect remains a key barrier to real-world use.

Despite compelling evidence of benefit, adoption of SGLT2 inhibitors in HF-CKD is hampered by therapeutic inertia, safety concerns disproportionate to their true risk, and underuse in patients with advanced CKD who were underrepresented in clinical trials. Expanding implementation requires clinician education, patient counseling, and pragmatic trials at the extremes of kidney function, particularly in dialysis populations where data remain scarce.

MRAs

Steroidal MRAs such as spironolactone and eplerenone are established components of GDMT for HFrEF, with mortality benefits demonstrated in pivotal trials such as the Randomized Aldactone Evaluation Study (RALES) and the Eplerenone Post-Acute Myocardial Infarction Heart Failure Efficacy and Survival Study (EPHESUS) [86,87]. These agents counteract the deleterious effects of aldosterone on the heart and vasculature, including fibrosis, adverse remodeling, and vascular stiffness. Their use in patients with CKD, however, presents major challenges. The most significant barrier is the risk of hyperkalemia, which is substantially higher in CKD and amplified when MRAs are prescribed, as guidelines recommend, in combination with RAAS inhibitors [88]. The fear of precipitating life-threatening hyperkalemia often deters clinicians from initiating therapy or from titrating doses upward, leading to underuse despite proven survival benefits.

The gap between evidence and practice is particularly wide in patients with advanced CKD, where the risk-benefit balance remains uncertain. Even when MRAs are started, concerns about potassium monitoring, lab follow-up, and polypharmacy frequently result in premature discontinuation. Until more robust evidence emerges for safer use in this population, the central challenge remains overcoming hyperkalemia risk through proactive monitoring, emerging potassium binders, and careful patient selection to extend the well-established benefits of MRA therapy to patients with HF-CKD.

Diuretics and Ultrafiltration in Acute Decompensated Heart Failure (ADHF)

The primary goal in treating ADHF is rapid and effective decongestion. Intravenous loop diuretics remain first-line therapy, but diuretic resistance is a frequent and particularly difficult problem in patients with CRS. CKD reduces the tubular delivery of diuretics and triggers compensatory mechanisms that blunt natriuretic response, limiting efficacy. Sequential strategies or ultrafiltration (UF) may be considered when loop diuretics fail. UF provides a nonpharmacologic means of fluid removal. Still, its role in routine care is constrained by concerns about electrolyte imbalances, intravascular depletion, hypotension, and sustained declines in kidney function often labeled worsening renal function (WRF) [89]. These risks frequently limit its use to highly selected cases of refractory congestion.

Recent evidence offers practical alternatives. The ADVOR trial demonstrated that adding acetazolamide to loop diuretics in ADHF significantly improved decongestion compared with loop diuretics alone, providing a feasible approach to overcoming diuretic resistance in patients with concurrent CKD [90]. This supports a shift from escalating loop doses alone toward more physiological, multi-segment blockade of sodium reabsorption. The challenge in real-world practice is balancing the urgency of decongestion against the risk of further renal injury. Clinicians must navigate narrow therapeutic margins, deciding when to escalate pharmacologic therapy, when to consider UF, and how to interpret transient rises in creatinine that may not always signal true kidney damage.

Other Pharmacologic Therapies

Beyond the core components of GDMT, several additional agents are used in selected subgroups of HFrEF. Ivabradine lowers heart rate by inhibiting the I(f) current in the sinoatrial node and is prescribed for patients in sinus rhythm who remain tachycardic despite optimal beta-blockade [91]. Vericiguat, a soluble guanylate cyclase stimulator, targets the nitric oxide pathway and is indicated for high-risk patients with worsening HF despite GDMT [92]. Hydralazine and isosorbide dinitrate (H-ISDN) combination reduces afterload and preload. It is particularly recommended for Black patients with persistent symptoms, or as an alternative when RAAS inhibitors are not tolerated [93].

The major challenge with these therapies lies in the absence of robust data in patients with advanced CKD or those on dialysis. Most pivotal trials systematically excluded individuals with severe renal impairment, leaving substantial uncertainty about their safety, dosing, and true effectiveness in this population [94]. As a result, clinicians are often hesitant to prescribe these medications to patients with HF-CKD, despite potential benefit in carefully selected cases. Without CKD-specific data, real-world use of ivabradine, vericiguat, and H-ISDN remains inconsistent. Until dedicated studies address these gaps, treatment decisions must rely on individualized risk-benefit assessment and close monitoring, highlighting the need for pragmatic research in the cardiorenal population.

Therapies for Comorbidities and Other Agents

Management of patients with HF and CKD is further complicated by therapies for common comorbidities, where benefits and risks are often magnified by renal impairment. Statins are a cornerstone of atherosclerotic cardiovascular disease prevention; however, their value in patients undergoing dialysis remains controversial. Large randomized trials have shown little benefit in this subgroup, leaving uncertainty about whether initiating statin therapy improves outcomes in advanced CKD [95]. Anticoagulation is critical for stroke prevention in atrial fibrillation, but advanced CKD substantially increases bleeding risk. Balancing thromboembolic protection with bleeding hazards represents a persistent clinical dilemma, and the optimal choice between vitamin K antagonists and direct oral anticoagulants in patients undergoing dialysis remains unsettled [96,97].

Intravenous (IV) iron is frequently used to treat iron deficiency, which is highly prevalent and associated with worse symptoms and outcomes in HF. While iron repletion improves exercise capacity and quality of life, its impact on significant clinical outcomes, such as mortality and hospitalization, remains debated [98-100]. Glucagon-like peptide-1 (GLP-1) receptor agonists (e.g., semaglutide) and newer dual GLP-1 agonists (e.g., tirzepatide) are emerging as essential therapies for diabetes and obesity, with proven cardiovascular benefit. Their role in HF is evolving: semaglutide improves symptoms and function in HFpEF and obesity; however, its impact in HFrEF or non-obese HFpEF remains uncertain [101-103]. Digoxin, a traditional therapy for HFrEF, reduces hospitalizations but does not improve survival. In CKD, its use is constrained by a narrow therapeutic window and reduced renal clearance, which markedly increases the risk of toxicity [104].

These therapies highlight the challenges of comorbidity management in HF-CKD: limited trial evidence in advanced CKD, narrow therapeutic margins, and heightened risks that complicate otherwise standard therapies. Individualized treatment decisions and close monitoring are crucial until more evidence specific to CKD becomes available.

Device and Advanced Therapies

For patients with HFrEF who remain symptomatic despite optimal medical therapy, device-based and advanced interventions are considered. These include implantable cardioverter-defibrillators (ICDs) to prevent sudden cardiac death, cardiac resynchronization therapy (CRT) to improve symptoms and ventricular function, and left ventricular assist devices (LVADs) or cardiac transplantation for those with end-stage disease [13].

In the cardiorenal population, however, these strategies are fraught with challenges. Advanced CKD substantially increases the risk of non-arrhythmic mortality, reducing the relative benefit of ICDs. It also heightens the procedural risks of device implantation, including infection, bleeding, and vascular complications. For advanced therapies, such as LVADs and transplantation, significant CKD often represents a relative or absolute contraindication, as it worsens survival and post-procedural outcomes.

Coronary revascularization in patients with ischemic cardiomyopathy poses similar difficulties. CKD amplifies the risks of contrast-induced nephropathy with percutaneous coronary intervention and surgical complications with coronary artery bypass graft, while long-term prognostic benefit remains uncertain. As a result, the threshold for recommending revascularization in HF-CKD is higher, and decisions are highly individualized [105]. Device and advanced therapies highlight the limits of current management in HF-CKD: although potentially life-saving in selected patients, their benefits are offset by competing risks and reduced efficacy in advanced CKD. These uncertainties make patient selection, timing, and risk-benefit assessment particularly complex in real-world practice.

Non-pharmacologic and Lifestyle Interventions

Non-pharmacologic strategies are integral to the management of HF and CKD, but their application in patients with both conditions presents unique challenges. Dietary sodium restriction is fundamental in HF care. However, overly strict limits can conflict with the nutritional needs of CKD patients, many of whom struggle with poor appetite, protein-energy wasting, and uremic symptoms. Similarly, fluid restriction must be carefully individualized: inadequate restriction contributes to persistent congestion, while excessive restriction increases the risk of dehydration and acute kidney injury [40].

Exercise programs and cardiac rehabilitation improve functional capacity and quality of life in HF, but remain underutilized in CKD patients due to concerns about frailty, comorbidities, and the lack of tailored rehabilitation programs for this vulnerable group. Ultimately, the complexity of managing overlapping HF and CKD highlights the necessity for multidisciplinary care, encompassing cardiologists, nephrologists, dietitians, and allied health professionals. However, in practice, coordination across specialities is often limited by logistical barriers, fragmented care systems, and inconsistent communication. Lifestyle and supportive interventions, while low-cost and potentially high-yield, are difficult to implement consistently in HF-CKD. The challenge lies in balancing nutritional adequacy with fluid and sodium control, adapting exercise safely, and building effective collaborative care models that can realistically support patients in everyday practice.

Polypharmacy, Deprescribing, and Patient-Centered Care

Managing multiple comorbidities with guideline-directed therapies often results in polypharmacy, which is particularly burdensome in patients with HF and CKD. It is not uncommon for individuals to be prescribed 10-15 or more medications, increasing the risk of drug-drug interactions, adverse events, and pill burden that erodes adherence and quality of life [106]. This creates a central dilemma: while guidelines strongly endorse the initiation and continuation of evidence-based therapies, there is a lack of evidence-based guidance on when and how to deprescribe medications that may be causing more harm than benefit. The lack of structured deprescribing protocols leaves clinicians uncertain about safely scaling back therapy in patients with advanced disease, frailty, or limited life expectancy [107,108].

Equally important is aligning treatment with patient goals. For many elderly or frail individuals with a high symptom burden, the priority may not be modest gains in long-term survival but rather improvement in functional capacity, symptom relief, and day-to-day quality of life. This requires shifting from a purely disease-centered model to one that emphasizes shared decision-making and the early integration of palliative care principles [109]. Polypharmacy highlights the tension between adhering to guidelines and tailoring care to individual needs. Without robust deprescribing evidence, clinicians must balance the risks of overtreatment with the need to honor patient preferences, making patient-centered care a critical yet often underdeveloped component of managing HF-CKD.

Clinical guidelines and evidence gaps

RAAS Inhibitors

Major guidelines from the American Heart Association (AHA), American College of Cardiology (ACC), Heart Failure Society of America (HFSA) (2022), European Society of Cardiology (ESC) (2021/2023), and Kidney Disease Improving Global Outcomes (KDIGO) (2024) consistently endorse RAAS inhibition as a foundation of therapy for HFrEF. ACE inhibitors or ARBs should be started with a “start low, go slow” titration, and a serum creatinine rise of up to 30% is considered acceptable to prevent premature discontinuation. Following PARADIGM-HF, guidelines recommend switching eligible patients from an ACE inhibitor/ARB to the ARNI sacubitril-valsartan to achieve greater reductions in morbidity and mortality [8,13,40,110].

This evidence gap has contributed to uncertainty and underrepresentation of this high-risk population in clinical practice. To address these gaps, guideline updates emphasize management of side effects rather than discontinuation. The KDIGO (2024) and AHA/ACC/HFSA (2022) recommend continuing ACE inhibitors/ARB/ARNI when clinically appropriate, with potassium binders and SGLT2 inhibitors available to mitigate hyperkalemia. Evidence from real-world studies and meta-analyses confirms that these strategies can enable the continuation and, in some cases, intensification of RAAS therapy in patients with HF-CKD [111-115]. The main barrier remains overcoming therapeutic inertia and fostering collaboration between cardiology and nephrology to ensure patients derive the proven benefits of guideline-directed therapy [116].

ARNIs represent the preferred RAAS therapy for many patients with HFrEF, but specific evidence in CKD is limited. Observational studies and meta-analyses suggest that sacubitril-valsartan improves cardiac biomarkers and function in mild-to-moderate CKD, though symptomatic hypotension often restricts dose escalation [117,118]. Evidence for kidney outcomes is inconsistent; recent meta-analyses do not confirm superiority over ACE inhibitors/ARB in preserving eGFR [119,120]. In patients with diabetes, ARNIs may also improve glycemic control and insulin sensitivity; however, these findings require further validation [121]. The most significant knowledge gap remains the absence of randomized controlled trial data in patients with eGFR <30 mL/min/1.73 m² or on dialysis, underscoring the need for dedicated trials to establish safety and efficacy in this high-risk group [122].

Beta Blockers

AHA and ESC guidelines recommend carvedilol, metoprolol succinate, or bisoprolol for all patients with HFrEF unless contraindicated, based on landmark trials (COPERNICUS, MERIT-HF, CIBIS-II) [13,40]. However, these trials excluded patients with advanced CKD (eGFR <30 mL/min/1.73 m²) and dialysis. Unlike RAAS inhibitors, where KDIGO provides renal-specific advice, no distinct recommendations exist for beta-blockers, reflecting their cardiology-centered focus. Evidence gaps and real-world findings highlight that the absence of CKD-specific trial data contributes to clinical uncertainty. Observational studies in patients with advanced CKD and dialysis show mixed outcomes: some report reduced mortality with beta-blocker use, while others found no clear survival benefit and noted increased risks of adverse effects, including hypotension, falls, and fractures. These findings underscore the challenge of applying trial-derived efficacy data to frail, multimorbid patients with cardiorenal disease. Need for tailored research and practice arises for the lack of randomized controlled trials in advanced CKD; clinicians must balance the proven survival benefit in mild-to-moderate CKD against the potential harms in late-stage disease. Individualized risk-benefit assessments and shared decision-making between cardiology and nephrology are essential until more robust evidence emerges [13,40].

SGLT2 Inhibitors

Current guidelines, along with large randomized trials, including DAPA-HF, EMPEROR-Reduced, DAPA-CKD, EMPA-KIDNEY, and subsequent meta-analyses, provide robust evidence that SGLT2 inhibitors reduce HF events, slow kidney disease progression, and lower mortality across diverse cardiorenal populations [82]. Reflecting on this, AHA/ACC/HFSA, ESC, and KDIGO guidelines now endorse SGLT2 inhibitors as foundational therapy for HFrEF and patients with albuminuric CKD, irrespective of diabetes status [13,40,110]. Trials in HFpEF (EMPEROR-Preserved, DELIVER) also demonstrated reductions in composite HF outcomes, leading to a class IIa recommendation in ACC/AHA/HFSA 2022 for use in selected HFpEF patients [13,40,110]. Overall, SGLT2 inhibitors represent one of the most consistent areas of concordance between cardiology and nephrology guidance.

Implementation gaps and outstanding questions remain a primary challenge, as ensuring that strong trial evidence is effectively translated into routine practice is often difficult. Compared with other drug classes, evidence gaps are narrower, but they remain most pronounced at the extremes of kidney function. Landmark trials enrolled patients to an eGFR of ~20-25 mL/min/1.73 m², and pooled analyses suggest benefits extend into this range. However, prospective randomized data are sparse for patients with eGFR <20 mL/min/1.73 m² and those on maintenance dialysis [123,124]. Mechanistically, benefits likely extend well beyond glucose lowering, including hemodynamic effects, natriuresis, tubular-metabolic modulation, and anti-inflammatory actions; however, further mechanistic and pragmatic studies are needed to optimize use in the sickest cardiorenal patients.

MRA

The AHA and ESC guidelines recommend steroidal MRAs (spironolactone, eplerenone) for patients with HFrEF, but with strict safety thresholds: avoid use if eGFR <30 mL/min/1.73 m² or if serum potassium >5.0 mmol/L. These restrictions limit use in the very population at highest risk. Introducing nonsteroidal MRAs, particularly finerenone, has partly addressed these challenges. In patients with CKD and type 2 diabetes, the FIDELIO-DKD, FIGARO-DKD, and pooled FIDELITY analyses demonstrated that finerenone reduces both kidney disease progression and cardiovascular events, with a lower risk of hyperkalemia compared with steroidal MRAs [13,40].

Integrating finerenone and expanding to HFpEF, the evidence base for MRAs is drawn from two distinct domains. HF trials of steroidal MRAs, such as spironolactone and eplerenone, demonstrated clear benefits in reducing morbidity and mortality but largely excluded patients with advanced CKD, limiting their generalizability to this high-risk group. Conversely, CKD trials of finerenone, a non-steroidal MRA with greater receptor selectivity and a more favorable safety profile, were not explicitly designed to evaluate HF outcomes. However, they did show significant renal and cardiovascular benefits. This divergence highlights both the promise and the gaps in evidence. At the same time, finerenone offers an opportunity to extend the benefits of MRA therapy into populations with impaired kidney function and potentially into HFpEF [13,40]. Robust, dedicated trials are still needed to confirm its role in these settings [13,40].

Recent trials are beginning to bridge this gap. The CONFIDENCE trial showed that combining finerenone with the SGLT2 inhibitor empagliflozin was superior to either drug alone in reducing kidney disease progression and cardiovascular events in CKD with type 2 diabetes [125]. Subgroup analyses of FIDELITY confirmed that finerenone reduces HF-related outcomes in CKD with diabetes [126]. Reflecting this, KDIGO (2024) guidelines now recommend finerenone in this population.

The role of MRAs in HFpEF remains unsettled. The TOPCAT trial of spironolactone produced inconsistent results across regions [110,127]. More recently, the FINEARTS-HF trial demonstrated that finerenone significantly reduced HF hospitalizations in patients with HFpEF and HFrEF. A renal outcomes analysis showed attenuation of eGFR decline, though without a significant effect on the primary kidney composite endpoint [128,129]. The largest evidence gap is the absence of large-scale randomized trials of any MRA in patients with HFrEF and advanced CKD (eGFR <30 mL/min/1.73 m²). The future challenge is to build on the improved safety profile of finerenone and its potential synergy with SGLT2 inhibitors to extend MRA benefits to the highest-risk cardiorenal patients. Table 2 represents foundational GDMT in HF.

**Table 2:** Foundational guideline-directed medical therapies in heart failure. AHA: American Heart Association; ACC: American College of Cardiology; HFSA: Heart Failure Society of America; ESC: European Society of Cardiology; KDIGO: Kidney Disease: Improving Global Outcomes; RAAS: renin–angiotensin–aldosterone system; HFrEF: heart failure with reduced ejection fraction; SGLT-2: sodium-glucose cotransporter-2; MRA: mineralocorticoid receptor antagonists; ACEI: angiotensin-converting enzyme inhibitors; ARB: angiotensin II receptor blockers; ARNI: angiotensin receptor-neprilysin inhibitors; CKD: chronic kidney disease; eGFR: estimated glomerular filtration rate; RCT: randomized clinical trial; HFpEF: heart failure with preserved ejection fraction; T2D: type 2 diabetes mellitus; UF: ultrafiltration; ICD: implantable cardioverter-defibrillators; CRT: cardiac resynchronization therapy. Table credit: Amaan Alvi References: [8,13,40,82,110-129]

Intervention	Guideline recommendation (AHA/ESC/KDIGO)	Primary evidence gap	Need for action
RAAS inhibitors (ACEi/ARB/ARNI)	Class I for HFrEF. ARNI is preferred over ACEi/ARB. KDIGO recommends albuminuric CKD. HFrEF & HFpEF → Class IIa	Lack of prospective RCT data in patients with advanced CKD (eGFR <30) and on dialysis.	Proactively manage hyperkalemia (e.g., with potassium binders or SGLT2 inhibitors) to enable its use. Conduct dedicated trials in the advanced CKD population.
Beta-blockers	Class I for HFrEF (carvedilol, metoprolol succinate, bisoprolol).	Lack of prospective RCT data in advanced CKD and dialysis. Observational data suggest potential for harm.	Critically re-evaluate the risk-benefit profile in frail, multimorbid patients. Conduct dedicated RCTs in the advanced CKD/dialysis population.
SGLT2 inhibitors	Class I for HFrEF and Class IIa for HFrEF/HFpEF, as per ACC/AHA/HFSA 2022. CKD: KDIGO 2024 recommends for adults with eGFR ≥20 mL/min/1.73 m² (albuminuria-specific grading); dialysis initiation not studied. Continue until dialysis or transplant if tolerated.	Limited prospective data in patients with eGFR <20 and those on maintenance dialysis.	Overcome clinical inertia to ensure widespread implementation. Conduct trials specifically targeting the populations with the lowest eGFR and those requiring dialysis. Consider combination with MRAs based on recent evidence for enhanced cardiorenal protection.
Mineralocorticoid receptor antagonists (MRAs)	Class I for HFrEF (with strict eGFR/potassium cutoffs). KDIGO recommends finerenone for T2D+CKD.	Lack of large-scale trials designed specifically for patients with concomitant HFrEF and advanced CKD (eGFR <30).	Finerenone has demonstrated cardiorenal benefit with a more favorable safety profile and may be considered in appropriate patients, although HF-specific outcome data are still emerging. Conduct dedicated trials of MRAs in the combined advanced HF-CKD population. Implement combination therapy with SGLT2i based on new evidence (e.g., CONFIDENCE trial).

Diuretics and Ultrafiltration in ADHF

The AHA, ESC, and KDIGO guidelines recommend intravenous loop diuretics as the first-line therapy for congestion in ADHF. However, these recommendations are primarily based on expert consensus, with limited trial data to guide the management of diuretic resistance, particularly in patients with advanced CKD. Traditional teaching discouraged thiazide-like diuretics in patients with eGFR <30 mL/min/1.73 m², citing reduced efficacy. UF is recognized as a potential option for refractory congestion, but its optimal role and timing remain debated [13,40,110].

Recent clinical trials have provided more substantial evidence for pharmacologic decongestion strategies. The CLICK trial showed that the thiazide-like diuretic chlorthalidone improves blood pressure and volume control in stage 4 CKD. The ADVOR trial demonstrated that adding acetazolamide to loop diuretics accelerates and enhances decongestion in ADHF [90,130,131]. These findings support a shift away from escalating loop diuretic doses alone toward a sequential nephron blockade strategy, which is more physiologic and effective. Although UF provides reliable fluid removal, evidence does not show a clear superiority over stepped pharmacologic therapy. The landmark CARESS-HF trial reported no significant benefit of UF compared with medical treatment and documented more adverse events, limiting enthusiasm for its widespread use [89,132].

The current consensus supports reserving UF for highly selected patients with refractory congestion who have failed optimized pharmacologic regimens [40,89,110]. The main evidence gap is identifying which patients with advanced CKD benefit most from UF versus intensified pharmacologic therapy. Until further trials clarify this, sequential nephron blockade should be prioritized, with use of UF reserved for selective cases.

Other Pharmacologic Therapies

Current HF guidelines for select HFrEF populations endorse several additional therapies. Ivabradine is recommended for patients with sinus rhythm with a heart rate ≥70 bpm despite maximally tolerated beta-blocker therapy. Vericiguat may be considered in high-risk patients with recent worsening HF events, based on its effects on the nitric oxide-soluble guanylate cyclase pathway. H-ISDN is specifically recommended for Black patients with persistent symptoms despite GDMT, or as an alternative when RAAS inhibitors are contraindicated. Data for these therapies in advanced CKD remain limited. In the SHIFT trial, ivabradine reduced HF hospitalizations and cardiovascular death, with consistent benefit across mild-to-moderate CKD, but very few patients had eGFR <30 mL/min/1.73 m² [133]. The VICTORIA trial of vericiguat included patients with eGFR ≥15 mL/min/1.73 m² but excluded those on dialysis; subgroup analyses suggested efficacy was maintained across renal strata [134].

Evidence for H-ISDN in advanced CKD is mixed. A large retrospective dialysis cohort reported lower all-cause mortality but paradoxically higher risks of HF hospitalization and myocardial infarction. These observational data are subject to residual confounding and cannot prove causality. Furthermore, in the era of modern GDMT, the incremental role of H-ISDN is unclear. Recent analyses also suggest that adding an SGLT2 inhibitor to H-ISDN in Black patients with advanced HF did not further reduce short-term readmissions [93,135,136]. For all three therapies, the absence of prospective, dedicated trial data in advanced CKD or dialysis populations remains the central limitation. Until such data are available, use in these groups must rely on individualized, case-by-case decision-making, balancing uncertain benefit against higher risks of adverse outcomes. Table 3 represents decongestion strategies and adjunct pharmacologic therapies in ADHF with CKD.

**Table 3:** Decongestion strategies and adjunct pharmacologic therapies in ADHF with CKD. Rows summarize guideline recommendations, main evidence gaps, and practical action points. Key trials referenced: ADVOR (acetazolamide + loop diuretic in ADHF), CLICK (chlorthalidone in Stage 4 CKD), CARESS-HF (ultrafiltration vs. stepped pharmacologic care). For each diuretic strategy, state eGFR where efficacy/safety is limited (units: mL/min/1.73 m²). Use sequential nephron blockade (loop + thiazide-like/acetazolamide) before UF in most cases; reserve UF for refractory congestion. Data and trial summaries were adapted from guideline texts and named trials. ADHF: acute decompensated heart failure; IV: intravenous; UF: ultrafiltration; eGFR: estimated glomerular filtration rate; CKD: chronic kidney disease; HF: heart failure; H-ISDN: hydralazine and isosorbide dinitrate. Table credit: Amaan Alvi References: [89,90,93,130-136]

Intervention	Guideline recommendation (AHA/ESC/KDIGO)	Primary evidence gap	Need for action
Diuretics & ultrafiltration (in ADHF)	Class I for managing congestion. Recommendations are primarily based on expert opinion.	Lack of data on long-term outcomes for combination diuretic strategies. The role of ultrafiltration is controversial.	Implement evidence-based sequential nephron blockade (e.g., as seen in ADVOR & CLICK trials). Reserve ultrafiltration for refractory cases.
Ivabradine, vericiguat, H-ISDN	Class IIa/IIb for specific, niche HFrEF populations.	Near-total absence of robust data in patients with advanced CKD or on dialysis.	Conduct prospective trials to define the safety, dosing, and efficacy of these agents in the severe cardiorenal population.

Therapies for Comorbidities and Other Agents

The guidelines emphasize the importance of addressing comorbidities in patients with HF-CKD, though evidence in advanced disease remains inconsistent. In the setting of anticoagulation, the RENAL-AF trial demonstrated that apixaban was not superior to warfarin for stroke prevention in patients with advanced CKD on dialysis, with similar bleeding risk, challenging the preferential use of direct oral anticoagulants (DOACs) in this population [97]. With respect to dyslipidemia, meta-analyses in dialysis cohorts found no reduction in cardiovascular mortality with statins, leading current guidelines to recommend against initiating statin therapy in patients receiving dialysis [137,138]. Iron deficiency represents another key comorbidity, where intravenous iron has consistently improved symptoms, yet the large HEART-FID trial did not show reductions in hard outcomes such as death or HF hospitalization [13,40].

For metabolic conditions, newer therapies such as GLP-1 receptor agonists (e.g., semaglutide) and dual GIP/GLP-1 agonists (e.g., tirzepatide) are increasingly recognized for their cardiovascular benefits in diabetes and obesity, with the STEP-HFpEF trial showing that semaglutide improved symptoms and functional capacity in obese patients with HFpEF, thereby positioning obesity itself as a therapeutic target. However, the comparative effectiveness of semaglutide versus tirzepatide and their potential role in HFrEF remain areas of active investigation [139]. Finally, digoxin continues to reduce HF hospitalizations without affecting mortality, but its use in CKD is limited by a narrow therapeutic window and an increased risk of toxicity due to impaired renal clearance [140].

Managing comorbidities in HF-CKD requires individualized decision-making. In end-stage kidney disease, anticoagulation and statin therapy should be guided by shared risk-benefit discussions. For intravenous iron, the focus should be on symptom relief rather than survival advantage. The role of GLP-1 receptor agonists across the HF spectrum, particularly in HFrEF, remains a major evidence gap. Likewise, the place of digoxin in the modern GDMT era, especially in advanced CKD, requires clarification through dedicated prospective studies. Table 4 summarizes the management of common comorbidities in HF-CKD.

**Table 4:** Management of common comorbidities in HF–CKD. AF: atrial fibrillation; DOAC: direct oral anticoagulant; ESKD: end-stage kidney disease; IV: intravenous; QoL: quality of life; ASCVD: atherosclerotic cardiovascular disease; HFpEF: heart failure with preserved ejection fraction; HF: heart failure; CKD: chronic kidney disease; eGFR: estimated glomerular filtration rate; HFrEF: heart failure with reduced ejection fraction; GLP-1: glucagon-like peptide-1; T2D: type 2 diabetes mellitus. Table credit: Amaan Alvi References: [97,137-140]

Intervention	Guideline recommendation	Primary evidence gap	Need for action
Statins	Recommended for ASCVD prevention. Not recommended for initiation in patients undergoing dialysis.	Uncertainty about the benefit of non-dialysis advanced CKD.	Highly individualized risk-benefit discussion with patients on dialysis.
Anticoagulants	Recommended for atrial fibrillation.	Uncertainty regarding the optimal agent (DOAC vs. warfarin) in end-stage kidney disease (ESKD).	Individualize decisions based on bleeding versus stroke risk, informed by recent trials such as RENAL-AF.
IV iron	Recommended for treating iron deficiency to improve symptoms and quality of life.	Lack of proven benefit on hard clinical outcomes (mortality, hospitalization) in recent large trials (e.g., HEART-FID).	Focus on using IV iron for symptom improvement rather than as a disease-modifying therapy for hard endpoints.
GLP-1 receptor agonists	Recommended for T2D/obesity. Proven benefit in HFpEF with obesity (e.g., semaglutide).	The role of HFrEF and non-obese HFpEF is undefined. The comparative effectiveness of different agents (e.g., versus tirzepatide) is under investigation.	Await results from ongoing, dedicated trials to define their role across the full spectrum of HF and CKD. Implement in HFpEF with obesity based on current evidence.
Digoxin	Not routinely recommended in guidelines; option for persistent symptoms	Reduces HF hospitalizations but not mortality	Narrow therapeutic index; reduced renal clearance in CKD → lower dose and therapeutic drug monitoring recommended; risk of toxicity elevated when eGFR is reduced (units: mL/min/1.73 m²).

Device and Advanced Therapies

The AHA and ESC guidelines strongly recommend ICDs for the primary prevention of sudden cardiac death and CRT for symptomatic improvement in appropriate HFrEF patients. For advanced HF, guidelines also endorse referral pathways for LVAD and transplant evaluation. However, these recommendations are based on pivotal trials that largely excluded patients with advanced CKD (eGFR <30 mL/min/1.73 m²) or those on dialysis. In clinical practice, significant CKD (e.g., irreversible eGFR <30-50 mL/min/1.73 m², depending on center criteria) is often regarded as a relative or absolute contraindication to LVAD implantation or cardiac transplantation [13,40].

Observational studies and meta-analyses suggest that ICD implantation rates are similar in CKD and non-CKD populations; however, the mortality benefit is attenuated or absent in dialysis patients [141]. In patients with advanced CKD and those receiving dialysis, the effectiveness of device-based therapies must be interpreted within a competing risk framework in which non-arrhythmic causes of death account for a substantial proportion of mortality. Observational data suggest that in dialysis-dependent populations, particularly in primary prevention settings, the relative contribution of sudden arrhythmic death is attenuated by high competing risks such as progressive HF, infection-related mortality, and treatment-limiting decisions related to overall disease burden and goals of care [13,40,141]. These factors reduce the potential absolute benefit of ICDs despite similar implantation rates compared with non-CKD populations. For CRT, available evidence, largely derived from observational studies and subgroup analyses, suggests that selected patients with HF and advanced CKD may experience symptomatic improvement; however, robust data on long-term outcomes such as survival and HF hospitalization in dialysis patients remain limited and inconsistent [13,40]. This uncertainty reflects the absence of randomized trials, reliance on heterogeneous registries, and potential confounding related to patient selection, HF phenotype, and severity of renal disease. Consequently, decisions regarding ICD and CRT implantation in advanced CKD require careful individualized assessment that integrates arrhythmic risk, competing modes of death, functional status, and patient preferences, rather than extrapolation from non-CKD trial populations. These uncertainties underscore the need for dedicated randomized clinical trials (RCTs) or robust registries to guide patient selection. Until such data are available, decisions regarding device therapy in advanced CKD must be individualized, balancing uncertain benefit against procedural risk.

Guidelines recommend a tailored approach to revascularization in patients with ischemic HF. However, recent systematic reviews and meta-analyses in HF-CKD cohorts found that percutaneous coronary intervention (PCI) or coronary artery bypass grafting (CABG) did not significantly improve long-term mortality or major adverse cardiovascular events compared with optimal medical therapy [105,142]. These findings reinforce the importance of optimizing GDMT before invasive strategies. Revascularization may be best reserved for patients with persistent symptoms or specific indications, pending future RCTs to clarify which CKD subgroups might derive meaningful benefit.

Non-pharmacologic and Lifestyle Interventions

The AHA, ESC, and KDIGO guidelines universally emphasize non-pharmacologic strategies as a core component of HF-CKD management. These include dietary sodium restriction, patient self-management, and promotion of physical activity. However, the supporting evidence in advanced HF-CKD is limited and often conflicting. For example, sodium restriction is fundamental in HF care, yet overly strict restriction in CKD may precipitate malnutrition and dehydration. Similarly, while exercise improves functional capacity in HF, practical recommendations for frail patients with significant cardiorenal disease remain poorly defined [13,40,110].

Emerging studies underscore the benefit of multidisciplinary care models integrating cardiology, nephrology, and dietetics, showing improved adherence to GDMT and better patient outcomes [143]. Despite this, substantial evidence gaps remain. In particular, no large RCTs are comparing different levels of sodium or fluid restriction, leaving the optimal dietary strategy uncertain.

Cardiac Rehabilitation and Physical Activity

Cardiac rehabilitation carries a class I recommendation for HF, but its safety, efficacy, and structure have not been adequately studied in patients with advanced CKD or on dialysis. Tailored rehabilitation programs are needed to accommodate the frailty and functional limitations unique to this population. Non-pharmacologic care remains foundational, yet most current recommendations are based on expert opinion rather than robust trial evidence. High-quality studies on diet, fluid management, and exercise in advanced CKD are essential to elevate these interventions to the same level of proof as pharmacologic therapies [21,70,144]. Table 5 summarizes device therapies, revascularization, and non-pharmacologic strategies in HF-CKD.

**Table 5:** The table represents device therapies, revascularization, and non-pharmacologic strategies in HF–CKD. AHA: American Heart Association; ESC: European Society of Cardiology; KDIGO: Kidney Disease: Improving Global Outcomes; ICD: implantable cardioverter-defibrillator; CRT: cardiac resynchronization therapy; LVAD: left ventricular assist device; HFrEF: heart failure with reduced ejection fraction; CKD: chronic kidney disease; RCT: randomized control trial; GDMT: guideline-directed medical therapy; HF: heart failure. Table credit: Amaan Alvi References: [21,70,105,141-144]

Intervention	Guideline recommendation (AHA/ESC/KDIGO)	Primary evidence gap	Need for action
ICD/CRT	Class I for eligible HFrEF patients.	Benefit is significantly attenuated or absent in patients on dialysis due to high competing risks.	Critically re-evaluate the risk-benefit ratio in individual patients with advanced CKD. There is a need for prospective registries or RCTs in this population.
Coronary revascularization	Individualized approach for ischemic HF.	Recent meta-analyses show no mortality benefit over medical therapy in non-dialysis-dependent CKD with ischemic heart disease; evidence in dialysis or mixed HF phenotypes remains limited.	Prioritize and fully optimize GDMT before considering an invasive strategy. Reserve for refractory symptoms.
LVAD/cardiac transplant	Referral pathway for advanced HF.	Significant CKD is a major relative or absolute contraindication.	Develop more effective criteria for patient selection and strategies to manage or mitigate renal dysfunction before and after the procedure.
Lifestyle & multidisciplinary care	Class I recommendation as foundational care.	Lack of robust RCTs on specific dietary or exercise protocols in the advanced HF-CKD population.	Build a high-quality evidence base for these foundational interventions. Prioritize the implementation of integrated, multidisciplinary care models that encompass cardiorenal care.

Polypharmacy, Deprescribing, and Patient-Centered Care

Polypharmacy is a pervasive challenge in patients with coexisting HF and CKD, reflecting the accumulation of guideline-directed therapies for multiple comorbid conditions. While contemporary HF guidelines emphasize initiation and titration of evidence-based therapies, explicit algorithms for deprescribing are limited, particularly in frail patients with advanced CKD [13,40,106]. Nevertheless, existing guidance does provide implicit hierarchies for therapy modification based on blood pressure, renal function, electrolyte disturbances, symptom burden, and treatment tolerance, as well as palliative care frameworks that support goal-concordant de-escalation in advanced disease [107-109]. The principal challenge lies not in a complete absence of guidance, but in translating these recommendations to heterogeneous HF-CKD populations with fluctuating volume status, recurrent hospitalization, and competing risks.

Importantly, pharmacologic deprescribing and device-related decision-making represent distinct clinical domains. Withdrawal or dose reduction of medications such as beta-blockers or RAAS inhibitors is driven by physiological tolerance, adverse effects, and patient priorities, whereas decisions regarding ICD therapy involve considerations of competing modes of death, procedural risk, and patient preferences rather than pharmacologic toxicity alone [13,40]. Conflating these processes risks obscuring their differing ethical, regulatory, and clinical implications. In practice, both require careful reassessment as patients progress through stages of HF and kidney disease, particularly during transitions such as recurrent decompensation, increasing frailty, or initiation of dialysis.

Patient-centered care is increasingly recognized as essential in HF-CKD, yet its implementation is complicated by prognostic uncertainty, discordance between clinician-estimated and patient-perceived benefit, and challenges in interpreting patient-reported outcomes in the setting of dynamic congestion and renal dysfunction [145-147]. While shared decision-making and early palliative care integration are widely endorsed, evidence to guide timing and operationalization in cardiorenal populations remains limited. Patient-reported outcome measures have potential value beyond descriptive endpoints, but their optimal domains and role in guiding therapeutic decisions in HF-CKD are not well defined. Addressing these gaps will require targeted studies that link deprescribing strategies, symptom burden, functional status, and quality of life, rather than survival alone, to better align treatment intensity with patient goals across the cardiorenal continuum.

A key priority is adopting a patient-centered approach. This includes the early integration of palliative care and shared decision-making, which aligns treatment with patient goals, often emphasizing symptom relief and quality of life over marginal survival benefits. Research is needed to establish and validate deprescribing protocols tailored for cardiorenal patients. Equally, future clinical trials should elevate patient-reported outcomes (PRO) to key secondary or even primary endpoints, ensuring that therapies extend survival and meaningfully improve daily function and well-being. Table 6 represents polypharmacy, deprescribing, and patient-centered care in HF-CKD.

**Table 6:** Polypharmacy, deprescribing, and patient-centered care in HF–CKD. AHA: American Heart Association; ESC: European Society of Cardiology; KDIGO: Kidney Disease: Improving Global Outcomes; ICD: implantable cardioverter-defibrillator; CKD: chronic kidney disease; HF: heart failure. Table credit: Amaan Alvi References: [145-147]

Intervention	Guideline recommendation (AHA/ESC/KDIGO)	Primary evidence gap	Need for action
Polypharmacy & deprescribing	Universally acknowledged as a challenge, but specific guidance is lacking.	No large-scale trials to guide safe deprescribing of therapies like beta-blockers or ICDs in frail patients.	There is an urgent need for research to develop and validate safe deprescribing protocols for the cardiorenal population.
Palliative care & shared decision-making	Universally endorsed as a principle of good care.	Lack of evidence on how to best incorporate patient-reported outcomes into routine management.	Shift the focus to a more patient-centered approach. Integrate palliative care principles early to align treatment with patient goals.

Table 7 highlights convergence across societies on the core pillars of HF therapy, i.e., RAAS inhibitors, beta-blockers, SGLT2 inhibitors, and MRAs, while outlining areas where recommendations diverge, particularly in the context of CKD. Notably, most landmark trials excluded patients with advanced CKD (eGFR <30 mL/min/1.73 m²) and dialysis, creating uncertainty regarding safety, efficacy, and optimal implementation in these populations. The evidence gaps column emphasizes unmet needs, ranging from hyperkalemia management with RAAS blockade, to limited RCT data for beta-blockers and SGLT2 inhibitors in late-stage CKD, to the evolving role of finerenone and MRAs in HFpEF.

**Table 7:** Comparative recommendations across AHA, ESC, and KDIGO guidelines for HF–CKD. AHA: American Heart Association; ACC: American College of Cardiology; HFSA: Heart Failure Society of America; ESC: European Society of Cardiology; KDIGO: Kidney Disease: Improving Global Outcomes; RAAS: renin–angiotensin–aldosterone system; HFrEF: heart failure with reduced ejection fraction; SGLT-2: sodium-glucose cotransporter-2; MRA: mineralocorticoid receptor antagonists; ACEI: angiotensin-converting enzyme inhibitors; ARB: angiotensin II receptor blockers; ARNI: angiotensin receptor-neprilysin inhibitors; CKD: chronic kidney disease; eGFR: estimated glomerular filtration rate; RCT: randomized clinical trial; HFpEF: heart failure with preserved ejection fraction; T2D: type 2 diabetes mellitus; UF: ultrafiltration; ICD: implantable cardioverter-defibrillators; CRT: cardiac resynchronization therapy. Table credit: Amaan Alvi References: [8,13,21,40,70,82,89,90,93,105,110-136,141-144]

Therapy	AHA/ACC/HFSA 2022	ESC 2021/2023	KDIGO 2024	Evidence gaps
RAAS inhibitors (ACEI/ARB/ARNI)	Class 1 for HFrEF; tolerate up to 30% rise in creatinine; switch ACEI/ARB → ARNI if eligible	Class 1 for HFrEF; recommend ARNI over ACEI/ARB	Recommended for albumin uric CKD; accept ≤30% creatinine rise	Advanced CKD (eGFR <30) & dialysis excluded from trials; hyperkalemia management strategies underused
Beta-blockers	Carvedilol, metoprolol succinate, bisoprolol for HFrEF (class 1)	Same three beta-blockers for HFrEF (Class I)	No CKD-specific guidance	Lack of RCTs in eGFR <30 or dialysis; mixed observational results
SGLT2 inhibitors	Class I for HFrEF; Class IIA for HFpEF	Class I for HFrEF; Class IIA for HFpEF	Strongly recommended for CKD with proteinuria, regardless of diabetes	Limited RCT data in eGFR <20 and dialysis; mechanisms beyond glycemia are still being clarified
MRAs	Spironolactone/eplerenone for HFrEF unless eGFR<30 or serum potassium >5.0 mmol/l	Same restrictions as AHA	Finerenone recommended for T2D + CKD; avoid steroidal MRAs if eGFR <30	No large RCTs of MRAs in HFrEF + advanced CKD; HFpEF evidence evolving (FINEARTS-HF)
Diuretics/decongestion	Loop diuretics first-line; consider sequential nephron blockade	Loop diuretics first-line; UF in refractory cases	Loop diuretics for volume control in CKD; thiazides may help at low eGFR	Few trials in advanced CKD; UF's role remains uncertain
Device therapy	ICD/CRT in appropriate HFrEF patients; advanced CKD is not specifically addressed	Similar to AHA	No formal guidance	Limited efficacy of ICD in dialysis; CRT evidence is inconsistent
Lifestyle and multidisciplinary care	Sodium restriction, exercise, and self-management are emphasized	Same	Same	Lack of large RCTs in advanced CKD for diet, rehab, or integrated clinics

Future directions

Future progress in the co-management of HF and CKD should prioritize four key translational goals. First, including patients with advanced CKD in HF randomized trials is essential to close critical evidence gaps and improve generalizability. Second, multimarker stratification strategies should be advanced, incorporating renal-independent markers such as sST2 (soluble suppression of tumorigenicity 2) alongside traditional biomarkers to capture risk across diverse patient populations better. Third, developing and implementing AI-assisted risk prediction tools that integrate biomarkers, imaging, and clinical variables could enable more precise and individualized care. Finally, expanding integrated cardio-renal clinics and multidisciplinary care models will be vital to address system-level fragmentation and ensure seamless management of this complex, high-risk population.

Risk Prediction and Biomarkers

Future advances in HF-CKD care require improved risk stratification strategies that address the limitations of existing clinical scores and single-biomarker approaches in this population. Conventional risk models often underperform in HF-CKD due to renal clearance effects on biomarkers, high biological variability, and frequent fluctuations in volume status and kidney function [148-162]. Multimarker strategies incorporating renal-independent biomarkers and serial measurements may improve prognostic accuracy, but their incremental value over optimized clinical assessment remains uncertain. Future research should prioritize comparative studies evaluating whether multimarker-based approaches meaningfully improve risk discrimination, therapeutic decision-making, or outcomes compared with standard clinician-guided care, rather than assuming additive benefit.

Artificial Intelligence (AI)

Artificial intelligence-based prediction tools offer potential advantages in integrating high-dimensional clinical and biomarker data, but their application in HF-CKD faces important domain-specific challenges. These include dataset bias arising from the systematic exclusion of advanced CKD patients from trials, confounding by treatment intensity, and instability of renal function over time [148-151]. While explainable AI frameworks are essential for clinical trust, future work must move beyond model development toward prospective validation and clinical impact assessment. Studies should determine whether AI-assisted strategies improve outcomes, reduce hospitalization, or enhance treatment safety beyond established risk-based approaches in HF-CKD populations.

Point-of-Care Testing (POCT) and Remote Monitoring

Point-of-care testing and remote monitoring technologies may shorten the feedback loop between physiological change and clinical intervention, but their utility in HF-CKD requires careful evaluation. Chronically elevated biomarkers, reduced renal clearance, and frequent biochemical variability increase the risk of false-positive alerts and overtreatment in advanced CKD [152-155]. Research priorities should include defining kidney-adjusted thresholds, identifying patient subgroups most likely to benefit, and evaluating whether intensified monitoring improves actionable decision-making without increasing patient burden or unnecessary intervention.

Implantable Hemodynamic Monitoring

Implantable hemodynamic monitoring has been shown to reduce HF hospitalizations in selected populations, yet its role in HF-CKD remains uncertain. Patients with advanced CKD face competing risks, higher procedural complication rates, and altered volume physiology that may modify the risk-benefit profile of implantable sensors [156,157]. Future studies should focus on patient selection, renal safety, and cost-effectiveness, with attention to whether hemodynamic-guided therapy offers incremental benefit over optimized clinical and biomarker-guided management in those approaching dialysis.

Imaging

Advanced imaging modalities, including strain echocardiography and non-contrast CMR imaging, offer opportunities to improve myocardial characterization while minimizing renal risk [158,159]. However, their clinical adoption is limited by uncertainty regarding outcome-linked validation, biological variability across CKD stages, and access constraints. Future research should prioritize studies that link imaging findings to therapeutic response and clinical outcomes, rather than descriptive phenotyping alone, and clarify where non-contrast imaging strategies can meaningfully inform management decisions in HF-CKD.

Laboratory Markers

Interpretation of conventional biomarkers such as BNP, NT-proBNP, and high-sensitivity troponins is complicated by renal clearance, anemia, and structural remodeling in CKD [160-162]. Establishing kidney-adjusted thresholds and validating serial measurement strategies represent important near-term priorities. Research should focus on whether such adjustments improve diagnostic accuracy, prognostication, or treatment guidance, rather than expanding biomarker panels without clear clinical utility.

Mechanistic Studies

Mechanistic research should prioritize kidney-driven pathways with direct therapeutic implications, including uremic toxins, inflammation, mineral metabolism, and anemia [162-165]. Rather than biomarker discovery alone, future studies should clarify whether these pathways can inform patient stratification for existing therapies or identify actionable targets for intervention. Integrating mechanistic substudies within clinical trials offers a pragmatic approach to linking biological insight with treatment response in HF-CKD.

Inclusive Trial Design

A persistent barrier to progress remains the exclusion of patients with advanced CKD from HF trials. Platform and registry-embedded trial designs that deliberately include CKD-heavy cohorts, with prespecified renal, biomarker, and imaging endpoints, are essential to address this gap [17,123-165]. Such designs would improve generalizability, enable subgroup analyses across CKD stages, and better inform real-world clinical decision-making.

Implementation Science

Despite growing recognition of multidisciplinary cardiorenal care models, uncertainty remains regarding which components drive outcome improvement. Future implementation research should move beyond integration as a general principle and focus on identifying high-impact elements, such as medication reconciliation, potassium management, or coordinated decongestion strategies, using pragmatic or stepped-wedge trial designs [8,40,110]. Clarifying these mechanisms is essential to translating evidence into scalable, effective care pathways for HF-CKD.

## Conclusions

The coexistence of HF and CKD represents a highly heterogeneous and clinically challenging spectrum in which overlapping hemodynamic, neurohormonal, inflammatory, and metabolic mechanisms interact differently across HF phenotypes and stages of kidney dysfunction. Although substantial progress has been made in understanding cardiorenal pathophysiology and in developing guideline-directed therapies for HF, patients with advanced CKD remain underrepresented in pivotal trials, limiting the certainty with which existing evidence can be applied to those at highest risk. Many associations central to cardiorenal disease, including congestion, biomarker elevation, uremic mediators, and anemia, are supported by robust observational data but incompletely established as causal, necessitating cautious interpretation and individualized clinical decision-making. Effective management, therefore, relies on an integrated, dynamic approach that balances decongestion, perfusion, renal stability, and patient goals rather than adherence to simplified mechanistic models. Future progress will depend on inclusive and mechanistically informed trial designs, refined risk stratification strategies, and pragmatic care models aligned with specific unresolved clinical decisions, thereby enabling more precise, patient-centered management of individuals living with both HF and CKD.
